# Genetically predicted HLA-DR+ natural killer cells as potential mediators in the lipid-coronary artery disease/ calcification (CAD/CAC) causal pathway

**DOI:** 10.3389/fimmu.2024.1408347

**Published:** 2024-08-29

**Authors:** Dingding Qian, Haoyue Zhang, Rong Liu, Honghua Ye

**Affiliations:** ^1^ Department of Cardiology, Lihuili Hospital Affiliated to Ningbo University, Ningbo University, Ningbo, Zhejiang, China; ^2^ School of Medicine, Ningbo University, Ningbo, Zhejiang, China

**Keywords:** Coronary Artery Disease, Mendelian randomization, Lipidome, immune cell, Natural Killer cell

## Abstract

**Background:**

Coronary artery disease (CAD) imposes a significant global health burden, necessitating a deeper comprehension of its genetic foundations to uncover innovative therapeutic targets. Employing a comprehensive Mendelian randomization (MR) approach, we aimed to explore the genetic associations between lipid profiles, immune cell phenotypes, and CAD risk.

**Methods:**

Utilizing data from recent large-scale genome-wide association studies (GWAS), we scrutinized 179 lipid and 731 immune cell phenotypes to delineate their genetic contributions to CAD pathogenesis, including coronary artery calcification (CAC). Moreover, specific immune cell phenotypes were examined as potential mediators of the lipid-CAD/CAC causal pathway.

**Results:**

Among the 162 lipid species with qualified instrumental variables (IVs) included in the analysis, we identified 36 lipids that exhibit a genetic causal relationship with CAD, with 29 being risk factors and 7 serving as protective factors. Phosphatidylethanolamine (18:0_20:4) with 8 IVs (OR, 95% CI, P-value: 1.04, 1.02-1.06, 1.50E-04) met the Bonferroni-corrected significance threshold (0.05/162 = 3.09E-04). Notably, all 18 shared lipids were determined to be risk factors for both CAD and CAC, including 16 triacylglycerol traits (15 of which had ≥ 3 IVs), with (50:1) exhibiting the highest risk [OR (95% CI) in CAC: 1.428 (1.129-1.807); OR (95% CI) in CAD: 1.119 (1.046-1.198)], and 2 diacylglycerol traits. Furthermore, we identified HLA DR+ natural killer cells (IVs = 3) as nominally significant with lipids and as potential mediators in the causal pathway between diacylglycerol (16:1_18:1) or various triacylglycerols and CAD (mediated effect: 0.007 to 0.013).

**Conclusions:**

This study provides preliminary insights into the genetic correlations between lipid metabolism, immune cell dynamics, and CAD susceptibility, highlighting the potential involvement of natural killer cells in the lipid-CAD/CAC causal pathway and suggesting new targets for therapy. Further evidence is necessary to substantiate our findings.

## Introduction

Coronary artery disease (CAD) remains a leading cause of global morbidity and mortality, necessitating continuous efforts to elucidate its complex pathogenesis and identify novel therapeutic targets. Dyslipidemia’s pivotal role in CAD development is well-established, but emerging evidence suggests that immune-inflammatory pathways, mediating lipid metabolism cascades, also play crucial roles in disease pathophysiology (1, 2). Understanding the intricate interplay between lipid metabolism and immune regulation may yield new insights into CAD mechanisms and pave the way for innovative therapeutic interventions.

Lipids play a central role in CAD onset and progression, with dyslipidemia recognized as a major risk factor for atherosclerosis and subsequent coronary events. Plasma lipids, typically measured through high-density lipoprotein cholesterol (HDL-C), low-density lipoprotein cholesterol (LDL-C), triglycerides, and total cholesterol, have been extensively studied (3, 4). Advanced lipidomic techniques have significantly expanded our understanding of circulating lipid variability and breadth (5, 6). Finer lipid classifications, such as cholesterol esters, ceramides, phosphatidylcholines, lysophosphatidylcholines, and diacylglycerols, offer potential improvements in CAD risk assessment (7). Additionally, ongoing advancements in omics technologies provide opportunities to identify genetic variants underlying a broad spectrum of lipid and immune cell phenotype determinants, potentially offering new intervention targets for cardiovascular diseases (8). However, specific lipid species’ potential impacts on CAD risk and the role of immune cells therein remain incompletely investigated.

In this study, we employed Mendelian randomization (MR) analysis to comprehensively investigate a wide array of 179 lipid profiles, assessing their genetic susceptibility to CAD and coronary artery calcification (CAC) (7). Additionally, we integrated genetic data from 731 immune cell phenotypes, utilizing a two-step MR analysis to evaluate their potential mediation in the lipid-CAD/CAC connection (9).

Our findings provide new insights into the complex genetic architecture of CAD and underscore the interdependence between lipid metabolism and immune cell dynamics in disease susceptibility. By revealing the genetic pathways linking lipids, immune cells, and CAD risk, our study offers new avenues for developing targeted therapeutic interventions aimed at mitigating CAD burden and improving patient prognosis. Importantly, this study employs an exploratory MR design, aiming not only to generate hypotheses and provide preliminary insights into the causal relationships between lipids, immune cells, and CAD/CAC but also to leverage genetic instruments to infer causality. This exploratory nature sets the stage for future research to build upon our findings and move toward more definitive conclusions.

## Methods

### Study overview

**Figure 1:**
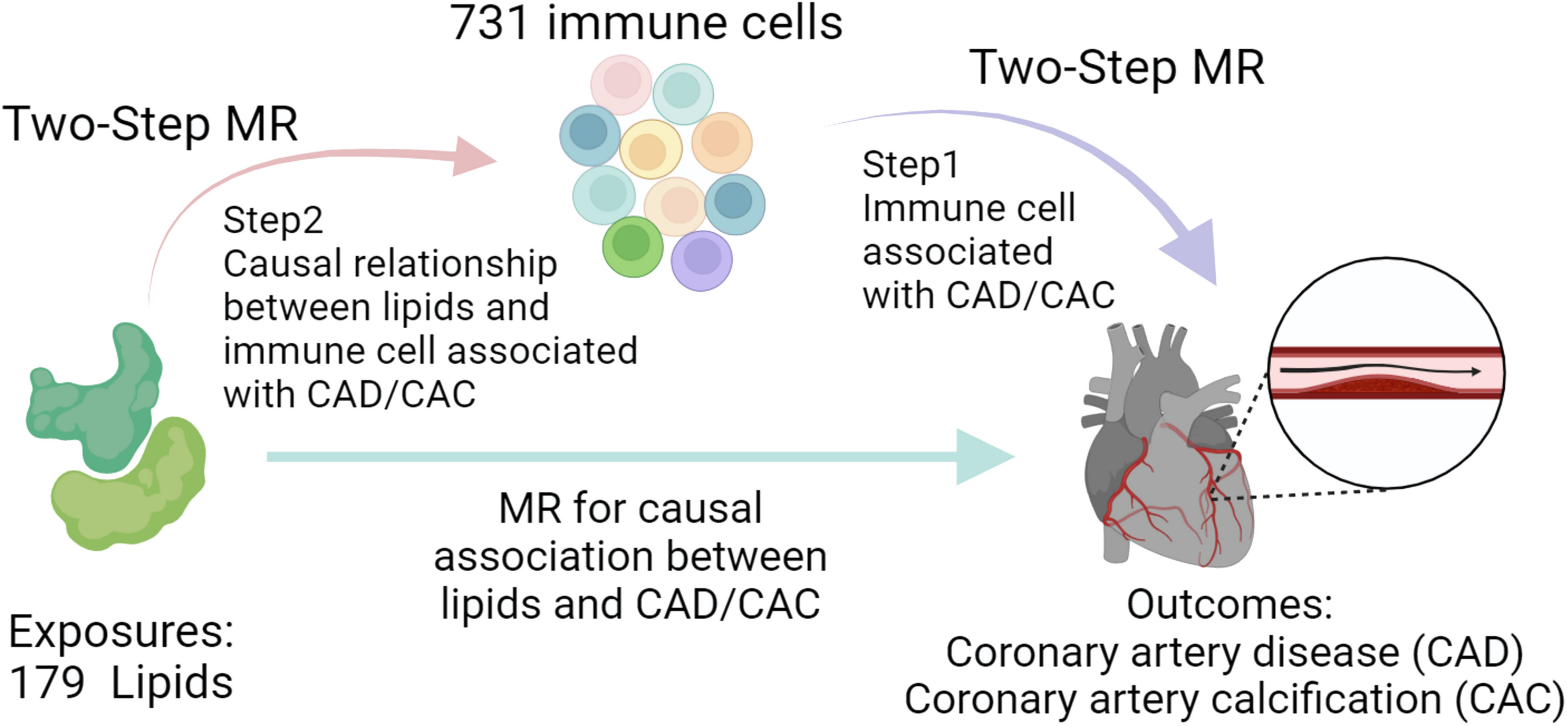
Study process overview. The study first conducts MR to assess the association between lipids and CAD/CAC, followed by a Two-Step MR approach. Step 1 identifies immune cell phenotypes associated with CAD/CAC, and Step 2 examines the causal link between these immune cells and lipid profiles, suggesting potential mediation pathways.

Our research aimed to uncover genetic factors associated with CAD by examining an expansive set of 179 lipid profiles, shedding light on deeper disease mechanisms and identifying new avenues for treatment. Drawing from a comprehensive lipidomic study, we extracted the respective genetic instrumental variables (IVs) related to lipid categories from 179 GWAS to evaluate their genetic predisposition to CAD through an MR approach. Additionally, we explored the genetic influence of significantly linked lipids on CAC risk. Our study also integrated recent genetic data on 731 immune cell phenotypes to assess the role of specific immune cells as potential intermediaries in the lipid-CAD/CAC connection, utilizing a two-step MR analysis (**Figure 1**). This comprehensive analysis aims to enhance our understanding of genetic underpinnings under CAD and pave the way for novel therapeutic interventions.

### Source of genetic information for included phenotypes

Our research utilized lipidomic data from a comprehensive analysis of 179 lipid species in a Finnish cohort, with datasets recorded in the HGRI-EBI Catalog (Study ID: GCST90277238 to GCST90277416) and detailed lipidome data accessible at https://www.ebi.ac.uk/gwas/publications/37907536 (7). GWAS of 179 lipidomes were batch downloaded and formatted in R software. We analyzed CAD using GWAS data from 181,522 cases among 1,165,690 participants, mainly of European descent, available at the Common Metabolic Diseases Knowledge Portal (CVDKP) (https://cvd.hugeamp.org/downloads.html#summary) (10). The latest CAC GWAS data, involving 26,909 Europeans, was also sourced from CVDKP (11). Additionally, we accessed GWAS data for 731 immune cell traits from 3,757 individuals from Sardinia via the IEU open GWAS database (https://gwas.mrcieu.ac.uk/), with access numbers ranging from ebi-a-GCST90001391 to ebi-a-GCST90002121 (9). All datasets comply with ethical guidelines from their respective studies, negating the need for further ethical approval for our secondary analysis.

### Genetic instruments of exposure

To ascertain causal connections between lipid and immune cell profiles (exposure) and CAD and CAC, we utilized exposed IVs as genetic proxies. Data processing was conducted primarily with the TwoSampleMR package (version 0.5.8) in R. We began by aligning the datasets to a common reference genome build (GRCh37) to ensure consistent genomic coordinates. Subsequently, all datasets were standardized, focusing on allele coding and effect size representation. We extracted relevant single nucleotide polymorphisms (SNPs) associated with lipid and immune cell phenotypes from GWAS summary statistics using the extract_instruments function. These SNP as IVs, serving as genetic surrogates, were selected based on their significant genetic correlations (P < 5 × 10^-8^) and minimal linkage disequilibrium (r² < 0.001) within a 10,000 kb span. Ensuring the ancestry of control and case samples was well-matched was critical to avoid confounding; traits without suitable IVs were excluded from the analysis. We excluded SNPs with F-statistic values under 10 to maintain instrument strength. Only phenotypes with SNPs meeting these criteria for correlation and independence were considered for analysis. True to the exclusion restriction principle for MR, our analysis focused on SNPs linked exclusively to lipid and immune cell levels, excluding empirically inferred potential confounding effects (smoking, alcohol consumption, hypertension, diabetes, BMI, lipid-lowering drugs, antihypertensives, and antidiabetic medications) on CAD/CAC risk using the NCBI’s LDlink tool by significant genetic correlations (P < 5 × 10^-8^) (12). The corresponding outcome data for the selected SNPs were extracted using the extract_outcome_data function, and the exposure and outcome datasets were harmonized with the harmonise_data function to align effect alleles (e.g., A/T, C/G). During the harmonization process (set the action = 2), palindromic variants with allele frequencies near 0.5 are typically excluded due to the challenge in accurately aligning them. Finally, we performed MR estimation and sensitivity analyses to validate the reliability of our findings.

### Statistical analysis

#### MR estimates of lipidome and CAD、CAC

To evaluate the causal impact of lipid profiles on CAD and CAC, our analysis utilized the TwoSampleMR package in R (version 4.3.1), applying the inverse variance weighting (IVW) method for variables with multiple genetic IVs and the Wald ratio for single-IV traits. The IVW method assumes all IVs are valid and non-interacting, suitable for complex variables. To enhance result reliability and minimize false positives, we employed the MR-Robust adjusted profile score (RAPS) technique from the mr.raps package (version 0.4.1), offering stabilized and accurate causal assessment through offering consistent and asymptotically normal estimators by adjusting the profile score to provide robustness to pleiotropy and weak instruments (13). To address the issue of multiple comparisons given the numerous lipids examined, we utilized the Bonferroni correction, which is a conservative method for reducing the likelihood of false positives. The adjusted significance threshold was set at P < (0.05 divided by the number of trait/SNP) and when the P-value does not reach the adjusted significance threshold but remains below 0.05, it is considered nominally significant. We aim to identify a broad range of potential associations, capturing initial signals that might be missed with stricter thresholds. The exploratory nature of this study is intended to generate genetic causal hypotheses, uncovering interesting patterns and associations for future, more targeted investigations. MR-Egger regression assessed pleiotropy in multi-IV analyses, with intercept P-values > 0.05 supporting result credibility.

#### Two-step MR estimates for immune cells as mediators in lipid-CAD/CAC association

Our investigation deepened the understanding of how lipid profiles potentially modulate CAD risk by affecting immune cell behaviors. Utilizing a two-step MR strategy (14), we initially pinpointed the direct influence of specific lipids on CAD, and CAC, and we derived common lipid types that potentially affect CAD and CAC. In the first step of MR, we determined which immune cell phenotypes were statistically significantly associated with CAD. In the second step, we can analyze the causal relationship between the lipids co-associated with CAC, CAD, and these specific immune cell phenotypes, shedding light on how lipid variations may predispose to CAD through immune modulation (**Figure 1**). Mediating candidates are recognized as logically consistent based on the direction of the MR effect. This dual-phase analysis underscored the interplay between lipid metabolism and immune cell dynamics, offering insights into novel genetic pathways that may underlie the lipid-immune influence on CAD risk. Significance thresholds were similarly corrected using 0.05 divided by the number of traits being compared.

## Results

### Lipids significantly causally associated with CAD

**Figure 2:**
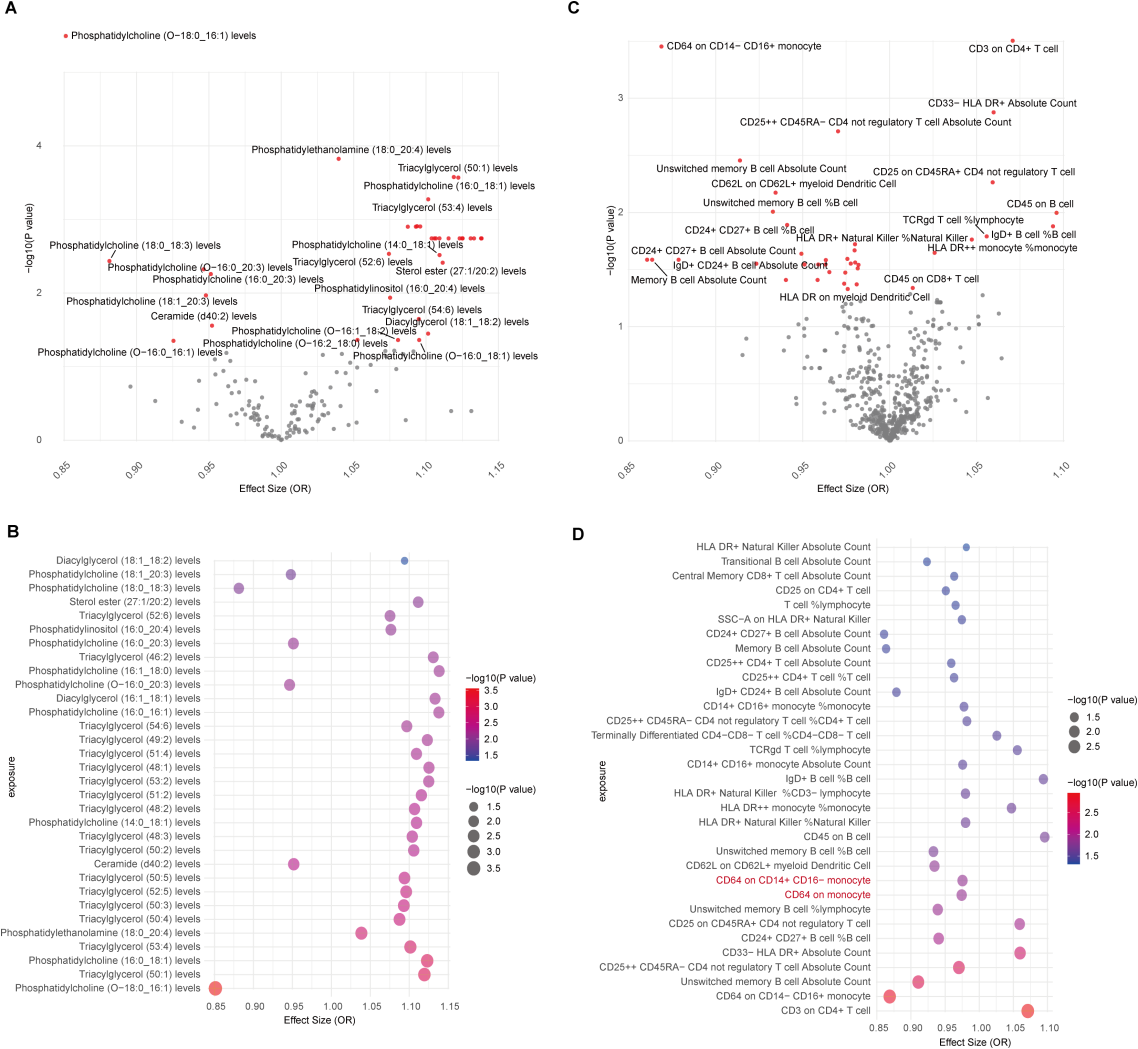
MR results for lipidomes, immune cell phenotypes, and CAD. **(A)** Volcano plot showing IVW/Wald ratio effect estimates between lipidomes and CAD, with significant lipid species highlighted in red; **(B)** Bubble plot displaying MR-RAPS estimates of significant lipids associated with CAD through IVW/Wald ratio estimation; **(C)** Volcano plot illustrating IVW/Wald ratio effect estimates between immune cells and CAD; **(D)** Bubble plot illustrating MR-RAPS estimates of significant immune cell phenotypes associated with CAD through IVW/Wald ratio estimation. Two immune cell types with horizontal pleiotropic bias are highlighted in red.

The causal association between lipids and CAD was investigated in our study. Initially, we identified the IVs for 179 lipid species, ensuring strong correlation and independence criteria were met. IVs for 162 lipids were successfully identified, with computed F-statistic values ranging from 29.79 to 1946.15, indicating no weak instrumental bias (**Supplementary Table 1**). Utilizing the IVW/Wald ratio for MR, we found that 36 lipids were genetically causally associated with CAD, with 29 acting as risk factors and 7 as protective factors (**Figure 2A**, **Supplementary Table 2**). This preliminary observation highlights a significant subset of lipid groups potentially influencing CAD risk. Specifically, Phosphatidylcholine (O-18:0_16:1) (OR, 95% CI, P-value: 0.85, 0.80-0.91, 3.21E-06), Phosphatidylethanolamine (18:0_20:4) (OR, 95% CI, P-value: 1.04, 1.02-1.06, 1.50E-04), Triacylglycerol (50:1) (OR, 95% CI, P-value: 1.12, 1.05-1.19, 2.65E-04), and Phosphatidylcholine (16:0_18:1) (OR, 95% CI, P-value: 1.12, 1.05-1.19, 2.70E-04) all met the Bonferroni-corrected significance threshold (0.05/162 = 3.09E-04) (**Supplementary Table 2**).

To corroborate the MR findings, robust RAPS analysis was conducted on the 36 lipids, revealing that 32 lipids passed the nominal significance threshold, including 26 risk factors (17 triacylglycerols, 4 Phosphatidylcholines, 2 Diacylglycerols, Sterol ester (27:1/20:2), Phosphatidylethanolamine (18:0_20:4) and Phosphatidylinositol (16:0_20:4)) and 6 protective factors (5 Phosphatidylcholines and Ceramide (d40:2)) (**Figure 2B**, **Table 1**, **Supplementary Table 3**). Elevated levels of 17 triacylglycerols were positively correlated with increased CAD risk (Odds Ratio, OR > 1). Importantly, the findings regarding finely categorized phosphatidylcholine traits, with 5 specific phosphatidylcholines exhibiting protective association against CAD (OR < 1). Conversely, 4 specific phosphatidylcholines were identified as strongly associated with elevated CAD risk, with phosphatidylcholine (16:1_18:0) demonstrating the highest risk (OR: 1.138, 95% CI: 1.036-1.251, P = 0.007) (**Figure 2B**, **Table 1**, **Supplementary Table 3**). To address potential directional horizontal pleiotropy bias in the MR results, Egger’s intercept test was performed for phenotypes with more than three IVs. The P values of the five MR estimate consistently exceeded 0.05, indicating an absence of bias by pleiotropy and thus enhancing the validity of our causal inferences (**Table 2**, **Supplementary Table 4**).

**Table 1:** Robust adjusted profile score (RAPS) estimates for lipidomes found to be significantly causally associated with coronary artery disease (CAD) by the IVW/Wald ratio methods.

ID.exposure	Trait.exposure	nsnp	Beta	P value	OR	OR_LCI	OR_UCI
GCST90277248	Sterol ester (27:1/20:2) levels	1	0.106	0.010	1.111	1.026	1.204
GCST90277254	Ceramide (d40:2) levels	2	-0.049	0.004	0.952	0.920	0.985
GCST90277260	Diacylglycerol (16:1_18:1) levels	1	0.125	0.007	1.133	1.035	1.241
GCST90277262	Diacylglycerol (18:1_18:2) levels	3	0.090	0.047	1.094	1.001	1.195
GCST90277273	Phosphatidylcholine (14:0_18:1) levels	2	0.104	0.005	1.109	1.032	1.192
GCST90277278	Phosphatidylcholine (16:0_16:1) levels	1	0.129	0.007	1.138	1.037	1.249
GCST90277281	Phosphatidylcholine (16:0_18:1) levels	2	0.116	0.001	1.123	1.047	1.205
GCST90277286	Phosphatidylcholine (16:0_20:3) levels	2	-0.050	0.008	0.951	0.917	0.987
GCST90277292	Phosphatidylcholine (16:1_18:0) levels	1	0.130	0.007	1.138	1.036	1.251
GCST90277301	Phosphatidylcholine (18:0_18:3) levels	1	-0.126	0.012	0.881	0.798	0.973
GCST90277312	Phosphatidylcholine (18:1_20:3) levels	3	-0.053	0.016	0.948	0.908	0.990
GCST90277322	Phosphatidylcholine (O-16:0_20:3) levels	2	-0.055	0.007	0.946	0.909	0.985
GCST90277335	Phosphatidylcholine (O-18:0_16:1) levels	1	-0.161	2.79E-04	0.851	0.780	0.928
GCST90277348	Phosphatidylethanolamine (18:0_20:4) levels	8	0.038	0.002	1.039	1.014	1.064
GCST90277360	Phosphatidylinositol (16:0_20:4) levels	3	0.074	0.008	1.076	1.019	1.137
GCST90277380	Triacylglycerol (46:2) levels	1	0.123	0.008	1.131	1.033	1.238
GCST90277382	Triacylglycerol (48:1) levels	1	0.118	0.006	1.125	1.035	1.224
GCST90277383	Triacylglycerol (48:2) levels	1	0.101	0.005	1.107	1.032	1.187
GCST90277384	Triacylglycerol (48:3) levels	1	0.099	0.005	1.104	1.031	1.182
GCST90277386	Triacylglycerol (49:2) levels	1	0.116	0.006	1.123	1.033	1.221
GCST90277387	Triacylglycerol (50:1) levels	2	0.113	0.001	1.119	1.046	1.198
GCST90277388	Triacylglycerol (50:2) levels	1	0.101	0.005	1.106	1.031	1.185
GCST90277389	Triacylglycerol (50:3) levels	2	0.089	0.003	1.093	1.031	1.159
GCST90277390	Triacylglycerol (50:4) levels	2	0.084	0.003	1.087	1.030	1.149
GCST90277391	Triacylglycerol (50:5) levels	2	0.090	0.003	1.094	1.030	1.161
GCST90277393	Triacylglycerol (51:2) levels	1	0.109	0.005	1.116	1.033	1.204
GCST90277395	Triacylglycerol (51:4) levels	1	0.104	0.006	1.109	1.030	1.194
GCST90277399	Triacylglycerol (52:5) levels	2	0.092	0.003	1.096	1.032	1.164
GCST90277400	Triacylglycerol (52:6) levels	3	0.073	0.008	1.075	1.019	1.135
GCST90277401	Triacylglycerol (53:2) levels	1	0.118	0.006	1.125	1.035	1.223
GCST90277403	Triacylglycerol (53:4) levels	2	0.097	0.002	1.101	1.037	1.170
GCST90277407	Triacylglycerol (54:6) levels	2	0.092	0.006	1.097	1.026	1.172
GCST90277331	Phosphatidylcholine (O-16:2_18:0) levels	1	0.051	0.054	1.053	0.999	1.109
GCST90277328	Phosphatidylcholine (O-16:1_18:2) levels	1	0.078	0.059	1.081	0.997	1.171
GCST90277320	Phosphatidylcholine (O-16:0_18:1) levels	1	0.091	0.063	1.095	0.995	1.206
GCST90277319	Phosphatidylcholine (O-16:0_16:1) levels	1	-0.077	0.066	0.926	0.852	1.005

nsnp, number of single nucleotide polymorphism; OR, odds ratio; OR_LCI, Odds ratio 95% lower confidence interval; OR_UCI, Odds ratio 95% upper confidence interval.

**Table 2:** Directional horizontal pleiotropy in the causal association of lipid and immune cell groups with CAD as assessed by MR-Egger regression.

Exposure	ID.exposure	Trait.exposure	Egger_intercept	Standard error	P value
Lipids	GCST90277262	Diacylglycerol (18:1_18:2) levels	0.054	0.020	0.220
	GCST90277312	Phosphatidylcholine (18:1_20:3) levels	0.001	0.026	0.987
	GCST90277348	Phosphatidylethanolamine (18:0_20:4) levels	0.002	0.008	0.849
	GCST90277360	Phosphatidylinositol (16:0_20:4) levels	0.041	0.026	0.360
	GCST90277400	Triacylglycerol (52:6) levels	-1.77E-04	0.044	0.997
Immune cells	GCST90001987	CD64 on CD14+ CD16- monocyte	0.035	0.012	0.025
	GCST90002006	CD64 on monocyte	0.032	0.011	0.028
	GCST90001417	CD24+ CD27+ B cell %B cell	0.135	0.087	0.365
	GCST90001432	Unswitched memory B cell %lymphocyte	0.010	0.011	0.468
	GCST90001649	HLA DR+ Natural Killer %Natural Killer	-0.003	0.004	0.472
	GCST90001585	CD14+ CD16+ monocyte %monocyte	-0.006	0.006	0.524
	GCST90001511	CD25++ CD45RA- CD4 not regulatory T cell %CD4+ T cell	-0.016	0.018	0.543
	GCST90001580	CD14+ CD16+ monocyte Absolute Count	-0.006	0.007	0.555
	GCST90001650	HLA DR+ Natural Killer %CD3- lymphocyte	-0.003	0.005	0.582
	GCST90002077	SSC-A on HLA DR+ Natural Killer	-0.004	0.007	0.660
	GCST90001648	HLA DR+ Natural Killer Absolute Count	-0.002	0.009	0.807

### Immune cell phenotypes genetically associated with CAD

Given the well-established role of immune cells in CAD, we aimed to assess the causal association of 731 immune cell phenotype groups with CAD. Utilizing similar criteria as for lipid species, we identified IVs for these immune cell phenotypes. Out of 731 phenotypes, 614 species with eligible IVs were successfully identified, with calculated F-statistic values greater than 10 (29.85 to 5062.70) (**Supplementary Table 5**). MR results revealed that 38 immune cell phenotypes with nominal significance were genetically causally associated with CAD, with 9 showing positive correlations and 29 showing negative correlations (**Figure 2C**, **Supplementary Table 6**). Robust RAPS analysis of these 38 correlated immune cells confirmed significant associations for 33 phenotypes, including 25 negative and 8 positive correlations (**Figure 2D**, **Supplementary Table 7**). Assessment of pleiotropy using MR-Egger’s method identified 2 immune cell phenotypes (CD64 on monocyte and CD64 on CD14+ CD16- monocyte) with potential bias due to pleiotropy in their estimates of CAD (**Figure 2D**, **Table 2**, **Supplementary Table 8**). Consequently, 31 immune cell phenotypes (9 phenotypes with ≥3 IVs and CD24+ CD27+ B cell %B cell passed significance threshold correction) were ultimately identified as having a significant causal association with CAD, comprising 23 protective factors and 8 risk factors.

### Lipidome and immune cell phenotypes associated with CAC

CAC serves as a prominent pathological hallmark of CAD (11). Here we utilized the recently released CAC GWAS data to characterize potentially genetically associated lipid profiles and immune cell phenotypes. Initially, employing the IVW/Wald ratio, we identified 39 lipid types significantly associated with CAC (**Supplementary Table 9**). Subsequent robust RAPS analyses excluded 5 potentially non-significant lipids, leaving 34 lipids confirmed to have a robust causal relationship with CAC. Notably, 18 of these lipids also exhibited a causal relationship with CAD, underscoring their importance in CAD pathology. Importantly, all 18 shared lipids were identified as risk factors for both CAD and CAC, comprising 16 triacylglycerol traits (15 species with ≥ 3 IVs) with (50:1) representing the highest risk [OR (95% CI) in CAC: 1.428 (1.129-1.807); OR (95% CI) in CAD: 1.119 (1.046-1.198); P <0.05/3] and 2 diacylglycerol traits, including (18:1_18:2) [OR (95% CI): 1.212 (1.058-1.388), P<0.05/5] and (16:1_18:1) [OR (95% CI): 1.370 (1.055-1.780), P<0.05/2] (**Table 3**, **Supplementary Table 10**). Assessment of pleiotropy did not invalidate the aforementioned causal assessment (P > 0.05) (**Table 4**, **Supplementary Table 11**).

**Table 3:** RAPS estimation for lipidomes significantly causally associated with coronary artery calcification (CAC) by the IVW/Wald ratio methods.

ID.exposure	Trait.exposure	nsnp	Beta	P value	OR	OR_LCI	OR_UCI
GCST90277238	Sterol ester (27:1/14:0) levels	1	0.836	0.001	2.308	1.380	3.861
GCST90277244	Sterol ester (27:1/18:0) levels	8	0.269	0.055	1.309	0.994	1.723
GCST90277246	Sterol ester (27:1/18:2) levels	8	0.330	0.004	1.391	1.109	1.745
GCST90277257	Cholesterol levels	1	0.599	0.023	1.820	1.086	3.048
GCST90277258	Diacylglycerol (16:0_18:1) levels	1	0.320	0.054	1.377	0.995	1.905
GCST90277259	Diacylglycerol (16:0_18:2) levels	1	0.265	0.051	1.303	0.999	1.700
GCST90277260	Diacylglycerol (16:1_18:1) levels	2	0.315	0.018	1.370	1.055	1.780
GCST90277261	Diacylglycerol (18:1_18:1) levels	5	0.194	0.012	1.214	1.044	1.412
GCST90277262	Diacylglycerol (18:1_18:2) levels	6	0.192	0.006	1.212	1.058	1.388
GCST90277263	Diacylglycerol (18:1_18:3) levels	1	0.349	0.055	1.418	0.993	2.024
GCST90277362	Phosphatidylinositol (18:0_18:2) levels	7	0.098	0.047	1.103	1.002	1.215
GCST90277370	Sphingomyelin (d34:1) levels	6	0.283	0.008	1.328	1.077	1.637
GCST90277382	Triacylglycerol (48:1) levels	2	0.328	0.022	1.388	1.048	1.840
GCST90277383	Triacylglycerol (48:2) levels	2	0.285	0.022	1.329	1.042	1.696
GCST90277384	Triacylglycerol (48:3) levels	2	0.271	0.020	1.311	1.044	1.647
GCST90277386	Triacylglycerol (49:2) levels	2	0.308	0.020	1.361	1.051	1.763
GCST90277387	Triacylglycerol (50:1) levels	3	0.356	0.003	1.428	1.129	1.807
GCST90277388	Triacylglycerol (50:2) levels	2	0.264	0.018	1.302	1.047	1.619
GCST90277389	Triacylglycerol (50:3) levels	2	0.229	0.017	1.258	1.042	1.519
GCST90277390	Triacylglycerol (50:4) levels	2	0.224	0.018	1.251	1.039	1.506
GCST90277391	Triacylglycerol (50:5) levels	2	0.243	0.020	1.275	1.040	1.564
GCST90277393	Triacylglycerol (51:2) levels	2	0.259	0.016	1.296	1.050	1.600
GCST90277394	Triacylglycerol (51:3) levels	5	0.254	0.001	1.289	1.110	1.495
GCST90277395	Triacylglycerol (51:4) levels	2	0.237	0.016	1.267	1.046	1.535
GCST90277396	Triacylglycerol (52:2) levels	4	0.263	0.002	1.300	1.102	1.534
GCST90277397	Triacylglycerol (52:3) levels	6	0.199	0.002	1.220	1.075	1.385
GCST90277398	Triacylglycerol (52:4) levels	6	0.240	3.41E-04	1.272	1.115	1.450
GCST90277399	Triacylglycerol (52:5) levels	3	0.227	0.005	1.255	1.070	1.472
GCST90277400	Triacylglycerol (52:6) levels	3	0.191	0.033	1.211	1.016	1.443
GCST90277401	Triacylglycerol (53:2) levels	2	0.254	0.015	1.289	1.050	1.582
GCST90277402	Triacylglycerol (53:3) levels	6	0.175	0.015	1.191	1.034	1.372
GCST90277403	Triacylglycerol (53:4) levels	4	0.237	0.003	1.268	1.086	1.480
GCST90277405	Triacylglycerol (54:4) levels	6	0.167	0.014	1.182	1.035	1.349
GCST90277406	Triacylglycerol (54:5) levels	4	0.200	0.011	1.222	1.046	1.427
GCST90277407	Triacylglycerol (54:6) levels	3	0.224	0.023	1.251	1.031	1.519
GCST90277408	Triacylglycerol (54:7) levels	3	0.210	0.046	1.234	1.003	1.518
GCST90277411	Triacylglycerol (56:5) levels	4	0.152	0.046	1.164	1.002	1.352
GCST90277413	Triacylglycerol (56:7) levels	4	0.204	0.010	1.226	1.051	1.431
GCST90277416	Triacylglycerol (58:8) levels	1	0.347	0.055	1.415	0.993	2.017

**Table 4:** Directional horizontal pleiotropy in the causal relationship of lipid and immune cell groups with CAC as assessed by MR-Egger regression.

Exposure	ID.exposure	Trait.exposure	Egger_intercept	Standard error	P value
Lipids	GCST90277246	Sterol ester (27:1/18:2) levels	-0.009	0.047	0.850
	GCST90277261	Diacylglycerol (18:1_18:1) levels	-0.005	0.033	0.885
	GCST90277262	Diacylglycerol (18:1_18:2) levels	-0.005	0.030	0.871
	GCST90277362	Phosphatidylinositol (18:0_18:2) levels	0.022	0.030	0.494
	GCST90277370	Sphingomyelin (d34:1) levels	-0.009	0.040	0.831
	GCST90277387	Triacylglycerol (50:1) levels	0.003	0.068	0.974
	GCST90277394	Triacylglycerol (51:3) levels	0.020	0.034	0.602
	GCST90277396	Triacylglycerol (52:2) levels	0.019	0.036	0.646
	GCST90277397	Triacylglycerol (52:3) levels	0.002	0.028	0.953
	GCST90277398	Triacylglycerol (52:4) levels	0.017	0.029	0.591
	GCST90277399	Triacylglycerol (52:5) levels	0.002	0.042	0.966
	GCST90277400	Triacylglycerol (52:6) levels	-0.061	0.067	0.534
	GCST90277402	Triacylglycerol (53:3) levels	-0.017	0.030	0.604
	GCST90277403	Triacylglycerol (53:4) levels	0.011	0.034	0.783
	GCST90277405	Triacylglycerol (54:4) levels	-0.011	0.022	0.660
	GCST90277406	Triacylglycerol (54:5) levels	-0.006	0.033	0.881
	GCST90277407	Triacylglycerol (54:6) levels	-0.025	0.038	0.628
	GCST90277408	Triacylglycerol (54:7) levels	-0.027	0.088	0.814
	GCST90277411	Triacylglycerol (56:5) levels	-0.058	0.070	0.494
	GCST90277413	Triacylglycerol (56:7) levels	0.018	0.048	0.742
Immune cells	GCST90001508	CD25++ CD45RA+ CD4 not regulatory T cell %CD4+ T cell	-0.031	0.018	0.193
	GCST90001509	CD25++ CD45RA+ CD4 not regulatory T cell %T cell	-0.029	0.018	0.202
	GCST90001621	Natural Killer T Absolute Count	-0.030	0.031	0.392
	GCST90001558	Terminally Differentiated CD8+ T cell %CD8+ T cell	0.015	0.035	0.743
	GCST90001562	CD45RA+ CD8+ T cell %T cell	-0.003	0.037	0.945
	GCST90001665	CD28+ CD45RA+ CD8dim T cell %CD8dim T cell	-0.001	0.018	0.961
	GCST90001667	CD28+ CD45RA- CD8dim T cell %T cell	0.006	0.128	0.970

Regarding immune cells, MR results suggested that 23 immune cells were causally related to CAC (**Supplementary Table 12**), and RAPS estimates further confirmed the robustness of causality for 19 immune cells, including 9 negative and 10 positive correlations (**Supplementary Table 13**). MR-Egger estimation did not reveal the presence of horizontal pleiotropy (**Table 4**, **Supplementary Table 14**). Interestingly, the analysis of immune cells with CAC yielded only one duplicate finding that passed corrected significance, CD24+ CD27+ B cell %B cell, compared to the CAD analysis.

### Immune cell-mediated lipid-CAD/CAC causal pathway

In light of the potential for lipids to influence CAD through modulation of immune cell phenotypes, we employed a two-step MR approach to evaluate immune cell types as potential mediators of the lipid-CAD/CAC causal pathway. We specifically focused on the 18 immune cell phenotypes that are co-associated with both CAD and CAC, and MR-estimated them against the 31 immune cell phenotypes robustly associated with CAD.

**Figure 3:**
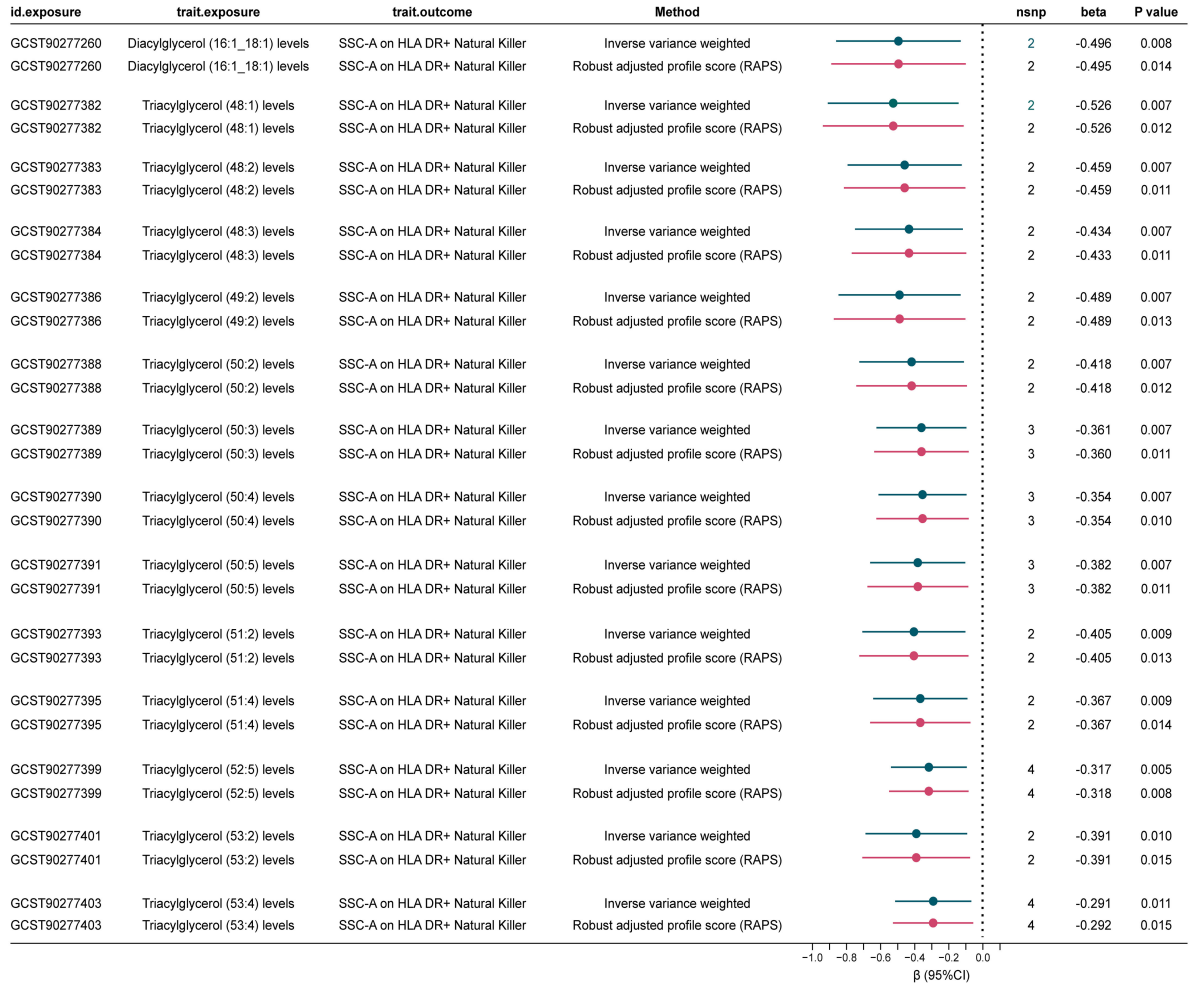
Significant mediated MR estimates for specific lipids and immune cell types in CAD. nsnp, number of single nucleotide polymorphism.

Our analysis revealed that 14 lipid levels, comprising 13 triglycerides and 1 diacylglycerol, were inversely genetically correlated with SSC-A on HLA DR+ Natural Killer cells (IVs = 3, β_IVW_: -0.291 to -0.526) at a nominal significant level (Corrected standard: P < 0.05/14 = 0.004). The RAPS analysis consistently confirmed the direction of causality estimation, bolstering the robustness of this mediation analysis (**Figure 3**, **Supplementary Table 15**). Further examination of the causal relationship between SSC-A on HLA DR+ Natural Killer cells and CAD demonstrated that lower levels of this immune cell were associated with higher CAD risk [OR (95%) = 0.975 (0.952-0.998), P = 0.034] (**Supplementary Table 6**). Collectively, these findings suggest that higher diverse triglyceride or diacylglycerol (16:1_18:1) levels are predictive of lower SSC-A on HLA DR+ Natural Killer cell levels, and lower SSC-A on HLA DR+ Natural Killer cells indicate higher CAD risk. Therefore, we identify SSC-A on HLA DR+ Natural Killer cells may play as a genetically predicted potential mediating immune cell in the lipid-CAD/CAC causal association pathway (mediated effect: 0.007 to 0.013).

## Discussion

The immunogenetics perspective underscores the intricate relationship among lipids, genes, and the immune system in atherosclerosis (15). Moreover, the interplay between lipid metabolism and immune function significantly impacts inflammation and immune response, influencing CAD development and progression (16). Benefitting from omics technology advancements and large-scale genetic data, our study explores the intricate interplay between lipid profiles, immune cell phenotypes, and the pathogenesis of CAD, offering fresh insights into potential mechanisms driving cardiovascular pathology. Beyond conventional lipidomics, our key findings not only underscored significant overlaps among lipid species associated with CAD and CAC but also elucidated potential mediating pathways linking lipid levels to CAD risk through the modulation of specific immune cell phenotypes.

Recent bioinformatics analyses identified distinct lipid metabolic patterns in CAD patients, suggesting unique characteristics within subgroups. Specific lipid metabolism-related genes were implicated in atherosclerosis progression, hinting at targeted genetic interventions (17). Our study identified 36 lipid traits causally associated with CAD and 4 passed the Bonferroni correction, comprising both risk and protective factors, underscoring the complexity of lipid metabolism in CAD pathogenesis. This finding is consistent with prior observational studies that emphasize the association of specific lipid species, such as various ceramides and phosphatidylcholines, with increased CAD risk (18–21). Previous research has identified specific ceramide subtypes, such as ceramide (d18:1), as predictors of adverse cardiovascular events in CAD (21). Our study expands this understanding by demonstrating that ceramide (d40:2) levels act as protective factors for both CAD and CAC.

Furthermore, 18 lipid types including 16 triacylglycerol traits (15 of which had ≥3 IVs) and 2 diacylglycerol traits were identified as shared risk factors for CAD and CAC, particularly emphasizing various triacylglycerols and diacylglycerols, supporting common underlying mechanisms driving arterial plaque calcification and underscoring the potential utility of CAC as a surrogate marker for stratifying CAD risk. For instance, triacylglycerol (50:1) exhibited the highest risk for both conditions, which is consistent with studies showing higher levels of triacylglycerols and diacylglycerols in myocardial infarction-prone rabbits compared to normal rabbits (19). In a gender-stratified lipidomic comparison study of CAD patients, women with CAD had lower diacylglycerols (18:1_22:4) and higher triacylglycerols (52:3) compared to those without CAD. In men with CAD, diacylglycerols (18:0_22:6), (16:0_16:0), (14:0_16:0), (16:0_18:0), and (16:1_18:0) were lower, while diacylglycerol (20:0_20:0) was higher compared to those without CAD (22). Our results identified nearly all diacylglycerols and triacylglycerols as risk factors for CAD or CAC. These discrepancies may be due to our focus on diacylglycerol (18:1), differences in lipid function based on fatty acid composition, methodological variations, and population heterogeneity. Interestingly, lipidomic analysis of patients with coronary microvascular dysfunction (MVD) showed lower concentrations of long-chain triacylglycerols and diacylglycerols, and higher concentrations of short-chain triacylglycerols. This contrasts with their traditional role as CAD risk factors and our findings, indicating specific pathobiological mechanisms in distinct diseases (23).

Emerging evidence suggests lipids profoundly influence immune cell function within atherosclerotic plaques, further shaping the inflammatory milieu (24–26). Our analysis elucidated the active involvement of immune cells in CAD pathogenesis, with 31 distinct phenotypes demonstrating significant causal relationships, indicating their integral role. We also identified immune cell phenotypes as potential mediators in the lipid-CAD/CAC pathogenic pathway. Through a two-step Mendelian randomization approach, we identified SSC-A on HLA DR+ Natural Killer cells as a potential mediator linking lipid levels to CAD risk, suggesting that lipid-induced alterations in NK cell activation status may contribute to CAD pathogenesis. We established a negative causal association of NK cells with CAD, consistent with reports of reduced NK cell levels in patients with CAD (27, 28). The expression of HLA-DR on NK cells suggests a more active and mature phenotype, and these cells may also have the ability to present antigens and interact with other components of the immune system (29). The interactions between NK cells and other immune cells, and their potential to present antigens due to the expression of HLA-DR, may imply that they play a more complex role in the immune response associated with CAD. These findings suggest that dysregulated lipid metabolism may not only directly lead to atherosclerosis but also indirectly influence CAD risk through NK cell dysregulation, opening avenues for exploring immune-targeted interventions as adjunct therapies in CAD management.

Our study contributes to understanding CAD pathophysiology by revealing complex interactions between lipid metabolism and immune cell function, emphasizing a potential CAD management approach. While the MR method offers advantages in establishing causal relationships and enhancing CAD risk prediction, several limitations may affect our results. Firstly, we applied a correction for multiple testing due to the large number of traits analyzed. This correction was necessary to reduce the risk of false positives, particularly given the exploratory nature of our study. The corrected significance thresholds resulted in some findings not meeting the adjusted criteria, underscoring the need for caution in interpreting these results. Secondly, given the exploratory nature of our analysis, we included all traits regardless of the number of IVs, taken to explore as many correlations as possible. Using a single IV in statistical analysis ensures consistency across different estimation methods, such as ratio and 2SLS, and allows valid testing of causal effects, even with a weak instrument. However, it faces challenges like the finite mean issue and potential bias from weak instruments, necessitating a P-value threshold of less than 0.03 for practical use (30). Therefore, caution should be exercised in interpreting these results and future GWAS with larger samples have more qualified IVs needed to support these results. Directional horizontal pleiotropy may confound causal inference, although MR-Egger regression tests indicate minimal bias (14, 31, 32). Furthermore, we addressed the genetic background of population stratification by mitigating weak instrumental bias using instrumental variables with high F-statistics and robust RAPS analyses for higher sensitivity validation of multicollinearity, as well as by focusing the dataset on participants of European descent only and applying genomic control methods (13, 33). It is important to acknowledge that while LDlink provided robust support for phenotype association screening of each IV, the exclusion restriction assumption based on the empirical exclusion of potential pleiotropic pathways has an element of subjectivity. This subjectivity may result in some unforeseen confounding factors not being entirely excluded, thereby impacting the precision of our findings. Although we employed various sensitivity analyses and rigorous statistical methods to enhance the robustness of our results, these inherent limitations could still influence our conclusions. Most GWAS including the studies we adopted have predominantly included individuals of European descent. This focus can limit the applicability of the results to other ethnic groups due to variations in genetic architecture and allele frequencies (32). Trans-ethnic GWAS are being conducted to incorporate a broader range of populations. Furthermore, considering the limitations of data selection is also necessary, such as potential discrepancies inherent to different datasets could influence our findings and the relatively small sample size of immune cells, a larger sample size may reveal more important associations that may not be detected in the current dataset, and thus further studies on larger immune cell datasets are needed to validate and extend our findings. Future research efforts should focus on elucidating the underlying mechanisms driving the observed associations, exploring potential therapeutic targets within the lipid-immune cell axis, and conducting clinical trials to assess the efficacy of immune-modulating therapies in CAD prevention and treatment. Integrating insights from lipidomics, immunology, and cardiovascular biology can deepen our understanding of CAD pathogenesis and facilitate personalized treatment strategies.

## Data Availability

The original contributions presented in the study are included in the article/**Supplementary Material**, further inquiries can be directed to the corresponding author/s.
